# Design and characterization of 3D printed, neomycin-eluting poly-L-lactide mats for wound-healing applications

**DOI:** 10.1007/s10856-021-06509-7

**Published:** 2021-04-08

**Authors:** Mahima Singh, Sriramakamal Jonnalagadda

**Affiliations:** Department of Pharmaceutical Sciences, Philadelphia College of Pharmacy, USciences 600 S 43rd St, Philadelphia, PA 19143 USA

## Abstract

This study evaluates the suitability of 3D printed biodegradable mats to load and deliver the topical antibiotic, neomycin, for up to 3 weeks in vitro. A 3D printer equipped with a hot melt extruder was used to print bandage-like wound coverings with porous sizes appropriate for cellular attachment and viability. The semicrystalline polyester, poly-l-lactic acid (PLLA) was used as the base polymer, coated (post-printing) with polyethylene glycols (PEGs) of MWs 400 Da, 6 kDa, or 20 kDa to enable manipulation of physicochemical and biological properties to suit intended applications. The mats were further loaded with a topical antibiotic (neomycin sulfate), and cumulative drug-release monitored for 3 weeks in vitro. Microscopic imaging as well as Scanning Electron Microscopy (SEM) studies showed pore dimensions of 100 × 400 µm. These pore dimensions were achieved without compromising mechanical strength; because of the “tough” individual fibers constituting the mat (Young’s Moduli of 50 ± 20 MPa and Elastic Elongation of 10 ± 5%). The in vitro dissolution study showed first-order release kinetics for neomycin during the first 20 h, followed by diffusion-controlled (Fickian) release for the remaining duration of the study. The release of neomycin suggested that the ability to load neomycin on to PLLA mats increases threefold, as the MW of the applied PEG coating is lowered from 20 kDa to 400 Da. Overall, this study demonstrates a successful approach to using a 3D printer to prepare porous degradable mats for antibiotic delivery with potential applications to dermal regeneration and tissue engineering.

**Figure:**
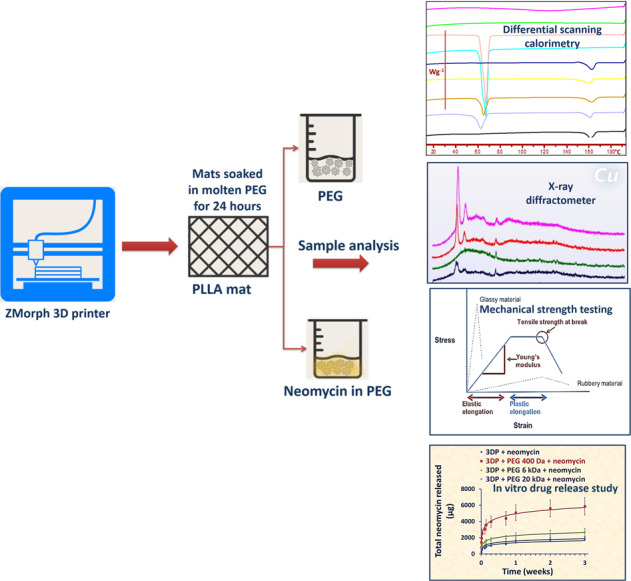
Illustration of the process used to create and characterize 3D printed PLLA mats.

Illustration of the process used to create and characterize 3D printed PLLA mats.

## Introduction

Approximately 6.5 million people in the United states suffer from chronic skin wounds, with costs exceeding $25 billion annually [1]. Superficial burns or wounds involve the outer epidermal layer the skin, and typically self-heal following the use of mild antiseptics. Partial thickness wounds are deeper, and may be further categorized into mid or deep, and involve the dermis [2]. Current treatment includes wound site debridement, followed by surgical interventions with autografts, allografts from cadavers, or xenografts typically from porcine sources. However, scarring, pain, availability of grafts and immune response to animal derived biomaterials warrants the need for additional research in wound care [3]. Significant progress has been made in the use of synthetic, semisynthetic, and natural biomaterials to treat skin wounds. Synthetic skin substitutes such as Biobrane^®^ [4] and TransCyte^®^ [5] are made from nondegradable silicone, and are useful as temporary wound closures. Currently available semisynthetic permanent skin substitutes such as Integra^®^ (polysiloxane, collagen, and glycosaminoglycan) have been clinically successful to treat skin wound [6]. These products often require extensive surgical attention and monitoring of the wounds subsequent to treatment. Additionally, the wound must be perturbed as the nondegradable polymer segments require removal following treatment. Commercially available grafts and dermal substitutes prepared from natural biomaterials such as collagen, chitosan, elastin, and glycosaminoglycan aim to mimic the functions of the extracellular matrix of the dermis [7]. Oasis wound matrix^®^ is a natural skin substitute made from epithelial cells in the intestinal lining [8]. Currently available skin substitutes are associated with high costs and most importantly; none of these products provide any antibacterial relief.

Wounds such as soft tissue, bite wounds, burn wounds, and diabetic foot ulcers are often infected by bacteria and aerobes such as *Staphylococcus aureus, Staphylococcus epidermidis, Pseudomonas aeruginosa, Staphylococcus aureus, Escherichia coli, Klebsiella, Enterococcus*, and *Candida* [9–11]. Infections in already traumatic wounds can further complicate 23% of high-risk wounds [12]. The primary outcomes for wound care is lowering the incidence of local and systemic infections [13], as well as eliminating secondary surgeries due to usage of nondegradable materials that may lead to further trauma [14]. Neomycin is an aminoglycoside antibiotic [15] with activity against gram negative bacteria and some gram positive bacteria commonly associated with topical infections. Neomycin is available in the form of creams, ointments, and eye drops [10, 16]. Recently, neomycin was loaded in a hydrogel dressing to study wound curing effects [17]. Neomycin-loaded nanofiber mats were prepared from poly (styrene sulfonic acid-co-maleic acid) (PSSA-MA) blended with polyvinyl alcohol (PVA) for use as wound dressing [18].

Poly-L-lactic acid (PLLA) [19], poly-caprolactone (PCL) [20], and poly-L-glycolic acid (PLGA) [21] are biodegradable, thermoplastic polymers that have been explored as sutures [22, 23], stents [24, 25], and nerve conduits [26]. These polymers have also been explored for tissue healing and replacement [27]. The thermoplastic nature of these polymers renders them amenable to melt-extrusion-based 3D printing [28]. PLLA is composed of monomers of lactic acid, which is biocompatible and well-tolerated, as it is a natural product produced by the human eccrine sweat gland [29]. The lactic acid contributes to a relatively lower skin pH of about 5, compared to the physiological pH of 7.4. PLLA is an aliphatic polyester that is manufactured from natural resources by a ring opening polymerization process [30]. The printing process parameters were recently explored in an effort to improve the mechanical characteristics of poly-lactic acid (PLA) [31]. Current techniques of producing wound dressings, films, and skin substitutes face processing limitations with respect to controlling pore sizes without compromising structural integrity [32]. These limitations may be readily overcome by additive manufacturing, which is an emerging area in pharmaceutical research [33, 34].

3D printing has been heavily explored to fabricate solid dosage tablets for oral delivery, [35] polypills, [36] controlled release formulations, [37] scaffolds, [38] and stents [39] for sustained drug release. The effects of geometry [40] and print angles [41] to characterize drug-release from oral dosage forms has also been studied. 3D printing in the field of pharmaceutical research has been deemed widely popular due to its ease to use, low costs, ability to replicate results, and providing a good platform for proof of concept studies. 3D printers allows the rapid variations in design parameters such as porosity and thickness, and is applicable for a wide range of thermoplastic polymers [42] and drugs [37]. The ease of use as well as adaptability allows for the transformation of the product easily for multiple applications [43]. However, 3D printing and drug releasing dermal dressings remain an unexplored area of research. The use of a 3D printer to print thermoplastic semicrystalline polymers such as PLLA subjects them to variable thermal stress, which can alter the balance between the amorphous and crystalline ratios of these polymers. Consequently, a thorough evaluation of the thermal, mechanical, and X-ray diffraction patterns will help elucidate process parameters to successfully prepare medical devices using this approach. The rate at which a semicrystalline polymer such as PLLA is treated during extrusion processes can affect its partial crystallinity. Annealing the semicrystalline polymer above its *T*_g_ can increase the amount of crystallinity of the material, while quenching can increase the amorphous content. In one such study, Wang et al. [44] studied the role of annealing on the mechanical properties of poly(lactic acid), and its blends with poly(3-hydroxybutyrate), prepared by extrusion-based additive manufacturing.

This research uses PLLA to prepare 3D-printed, neomycin eluting, dermal bandages to validate the utility of 3D printers over other conventional methods. 3D printed PLA:PEG scaffolds have been previously explored for the delivery of drugs such as dimethyloxalylglycine (DMOG), erythropoietin (EPO), prednisolone, and dexamethasone [45–48]. The method of drug incorporation is a critical factor in determining drug loading and release kinetics from loaded devices. While several methods exist for drug loading, our method employed a coating approach, post-manufacture of the 3DP mats. Drug loading onto polymeric fibers post-manufacture has been explored previously and relies on passive drug diffusion into swollen polymeric molecules [47]. The loading may be enhanced by increasing exposure time of the polymer to the drug solution [49]. In our study, a soaking method [47, 49] was used to load neomycin onto printed PLLA bandages (mats), and drug release was evaluated over a period of 3 weeks. All samples were analyzed for thermal and mechanical changes as well as studied for drug release in the presence of polyethylene glycols (PEG) of molecular weights (MW) 400 Da, 6 kDa, and 20 kDa. The inclusion of PEG [50] and other plasticizers such as polyhydroxyl butyrate (PHB) [44, 51] and polyvinyl acetate (PVA) [52, 53] has been reported previously.

## Materials and methods

### Materials

PLLA filament (1.75 mm, clear) was purchased from 3D Solutech, Seattle, WA. PEGs of MW 400 Da and 20 kDa were purchased from Fluka BioChemika, Seelze, Germany. PEG 6 kDa was purchased from Alfa Aesar, Thermo Fisher Scientific, MA, USA. Neomycin trisulfate salt hydrate, glacial acetic acid, acetylacetone ReagentPlus ≥99%, and formaldehyde 37% in water were purchased from Sigma-Aldrich, St Louis, MO. Aluminum pans (40 µL) and lids were purchased from Mettler Toledo, Columbus, OH.

### Methods

#### Mat preparation

A ZMorph 3D printer was used to print PLLA mats. Splicing and manufacturing parameters were optimized, and mats of dimensions 10 × 10 × 0.4 mm were printed with the parameters listed in Table 1.

**Table 1:** Values of parameters used in Voxelizer software to manufacture 3D printed mats

Printing parameter	Value
Nozzle diameter	0.2 mm
Nozzle temperature	200 °C
Print bed temperature	60 °C
Print bed material	Teflon tape on top of glass bed
Print speed	35 mm/s
Layer thickness	0.4 mm
Infill pattern	Rectilinear
Infill percentage	40%

Once printed, mats were stored in a sealed container containing silica gel desiccant. A soaking method described previously in literature [54] was used to incorporate the PEG. Briefly, the printed mats were heated to 65 ± 5 °C on a glass petri dish placed on a hot plate and cut into smaller dimensions of 1 × 1 × 0.4 mm. About 15 g of PEGs of MW 400 Da, 6 kDa, or 20 kDa were added to 20 mL scintillation vials and heated to 70 °C for 30 min to melt the PEG. The PLLA mats were then added to these PEG-containing scintillation vials, ensuring complete immersion. The vials were maintained at 70 °C for 24 h to ensure incorporation. After 24 h, mats were removed, washed by dipping in 70% ethanol, placed in fresh scintillation vials, and imaged for morphology. To prepare neomycin-containing mats, PLLA mats were added to PEG and neomycin-containing scintillation vials. These vials were prepared in a manner identical to those with PEG alone, except that 1 mL of a 2.5% stock solution of neomycin trisulfate was added to the PEG prior to PLLA submersion.

#### Morphological analysis

For morphological analysis, imaging studies were performed to generate magnified images of the 3D printed (3DP) mats using an optical microscope. Furthermore, in order to confirm porosity and surface characteristics, a scanning electron microscope was also used to image the mats.

##### Optical microscopy

A polarized optical microscope with a camera attachment, AmScope Microscope digital camera (MU1000 series) (CA, USA) was used. The samples were placed on the stage, images were taken using the camera, and analyzed with the AmScope software.

##### Scanning electron microscopy (SEM)

SEM analyses were performed using a FEI Quanta^TM^ 250 environmental scanning electron microscope (Thermo Fisher Scientific, Hillsboro, Oregon, USA). All samples were analyzed at varying magnifications. Samples were placed onto the conductive aluminum specimen stub with adhesive carbon discs. The stage with the film sample was raised at 10 mm working distance of the polar piece of the microscope, and images were taken from the surface with the voltage set at 10 kV.

#### Mechanical testing

**Fig. 1:**
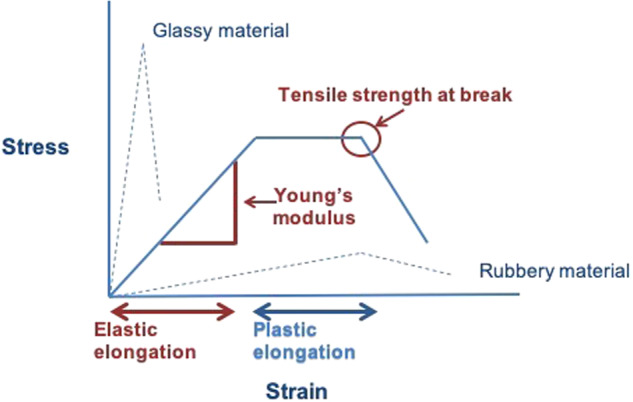
Illustration of the measured parameters for the mechanical testing experiments

A Shimadzu Autograph tensile tester (Shimadzu Scientific Instruments (SSI), Kyoto, Japan) supported with the Trapezium^®^ software and equipped with a 50 N load cell was used for mechanical analysis of individual filaments forming the PLLA mat. Fibers of dimensions 10 × 0.5 mm were immersed in molten PEG or neomycin-PEG blends, prepared in a manner similar to those described in Section “Mat preparation”. The fibers were secured between two vertically placed jigs, as the length of elastic elongation (EE) (strain) and the maximum force at break (stress) were recorded. The slope of stress vs. strain graphs was used to calculate Young’s modulus (Fig. 1).

#### Differential scanning calorimetry (DSC)

For DSC analysis, samples weighing ~5–10 mg were cut from the sample mats, placed in 40 µL aluminum crucible pans, and hermetically sealed with a crimping device. Nitrogen with a flow rate of 20 mL/min was used as a purge gas. For 5 min, all samples were held at 25 °C isothermal conditions, then subjected to two subsequent heating cycles with an intermittent cooling cycle. A heat/cool rate of 10 °C/min, and a temperature range of 25–200 °C was used for all samples.

DSC thermograms measure specific heat of materials as a function of heating temperature, and are used to study phase transitions in the solid state. Amorphous solids have a glass transition (*T*_g_), observed as a shift in the baseline. This shift corresponds to an increase in specific heat between a brittle/glassy and a flexible/rubbery physical state. At a molecular level, this change in specific heat (Δ*C*_p_) represents an increase in a cooperative, segmental motion of the polymeric chains. In contrast, crystalline solids do not exhibit a glass transition, and directly form a melt at the melting temperature (*T*_m_), observed as a peak on DSC thermograms. The associated melting enthalpy (Δ*H*_m_) is characteristic of the crystalline packing of the material, and may be used to quantitate the crystalline fraction in a semicrystalline polymer such as PLLA. The DSC thermograms of the samples were evaluated for the glass transition temperature (*T*_g_), cold crystallization temperature and enthalpy (*T*_c_ and Δ*H*_c_), as well as melting temperature and enthalpy (*T*_m_ and Δ*H*_m_). Percent crystallinity of the samples was calculated from Δ*H*_m_ values, using a literature value of 93.7 J/g [55] as a reference for 100% crystalline PLLA.

#### Powder X-ray diffractometry (PXRD)

PXRD analysis was performed using a Rigaku Miniflex X-ray Diffractometer (Rigaku Corp. Tokyo, Japan). A double-sided adhesive tape was applied over the sample holder, and powdered samples were layered on the tape using a thin spatula. The diffracted intensity was analyzed at a 2θ range at a starting angle of 10° and ending at 70°. A Cu anode tube (lambda (*λ*) = 1.54 Å) was used. All diffractograms were analyzed using the JADE software. The size of the crystallites, *L*_hkl_, was quantified using the Scherrer’s equation [56, 57] *L*_hkl_ = kλ/ B_2θ_*cos θ. The Miller indices, hkl, when perpendicular to the lattice planes, are used to analyze the crystallite size. The crystallite-shape factor, k, is a numerical factor and a value of 0.89 was used [58]. λ is the wavelength of the X-rays (cu = 1.54 Å), B_2θ_ is the full-width at half-maximum of the diffraction peak in radians and θ is the Braggs angle.

#### Drug-release analysis

##### Neomycin assay

Neomycin was assayed using a Hantzsch reaction, described previously in literature [59]. The following steps were used to prepare a “Hantzsch solution”. 10 ml of 0.6 M acetic acid and 0.8 mL acetylacetone were added to 1.5 mL of 40% formaldehyde and diluted to 30 mL using deionized (DI) water. The pH was adjusted to 2.5 using 1 M HCl. First, a stock solution of neomycin in DI water was prepared at 2.5% (w/v). Using the neomycin stock solution, a dilute solution of 0.01% neomycin was prepared as a standard. Finally, a 1:1 solution made from 2 ml of Hantzsch solution and 2 ml of standard were mixed, placed in a scintillation vial, capped and sealed using aluminum foil. The mixture was then placed in a boiling water bath at 100 °C for 20 min. After cooling to room temperature, 5 mL of DI water was added. A new stock solution of 10 µg/ml neomycin was prepared from the above reaction vial. Samples were prepared in serial dilutions of 1–100 µg/ml. A spectrum analysis was performed to check for λ max, and absorbance was taken at 356 nm using 200 µl of each sample.

##### Neomycin release

Triplicate samples of all PLLA mats were placed in 20 ml scintillation vials containing 20 ml DI water, maintained at 37 °C in a shaking water bath (40 rpm). Periodically, samples were removed at 0.5, 1, 2, 4, 18, 24, 48 h, and then weekly for 3 weeks. During each sampling period, 2 mL of sample was withdrawn, which was replaced by 2 mL of fresh, preheated DI water to maintain sink conditions. The dissolution samples were stored in 2 mL eppendorf tubes, at 4 °C. Drug content was analyzed using the Hantzsch reaction described previously. Cumulative drug-release profiles were plotted as a function of time. Drug release was analyzed for release rate, duration, and total amount of drug released.

#### Statistical analysis

All experiments were performed in triplicates (*n* = 3). To evaluate differences between mean values, analysis of variance (ANOVA) and a post hoc Tukey test were used. Statistical significance was established at *p* < 0.05. Drug-release studies were conducted in triplicate and data from the drug-release study was analyzed using Microsoft^®^ Excel to generate linear regression fits.

## Results

### Mat preparation and morphology

The 3D printed mats (3DP) consisted of uniformly layered, fused PLLA fibers with rectangular pores of pore sizes of roughly 0.1 × 0.4 mm. The large surface area enabled by the fibrous grid-structure could ensure cell and platelet adhesion to allow for clot formation, which is a preliminary step in the wound-healing cascade. The large porous structure is expected to ensure permeability to congealed plasma during this preliminary stage, thereby keeping the wound dry to facilitate fast healing.

**Fig. 2:**
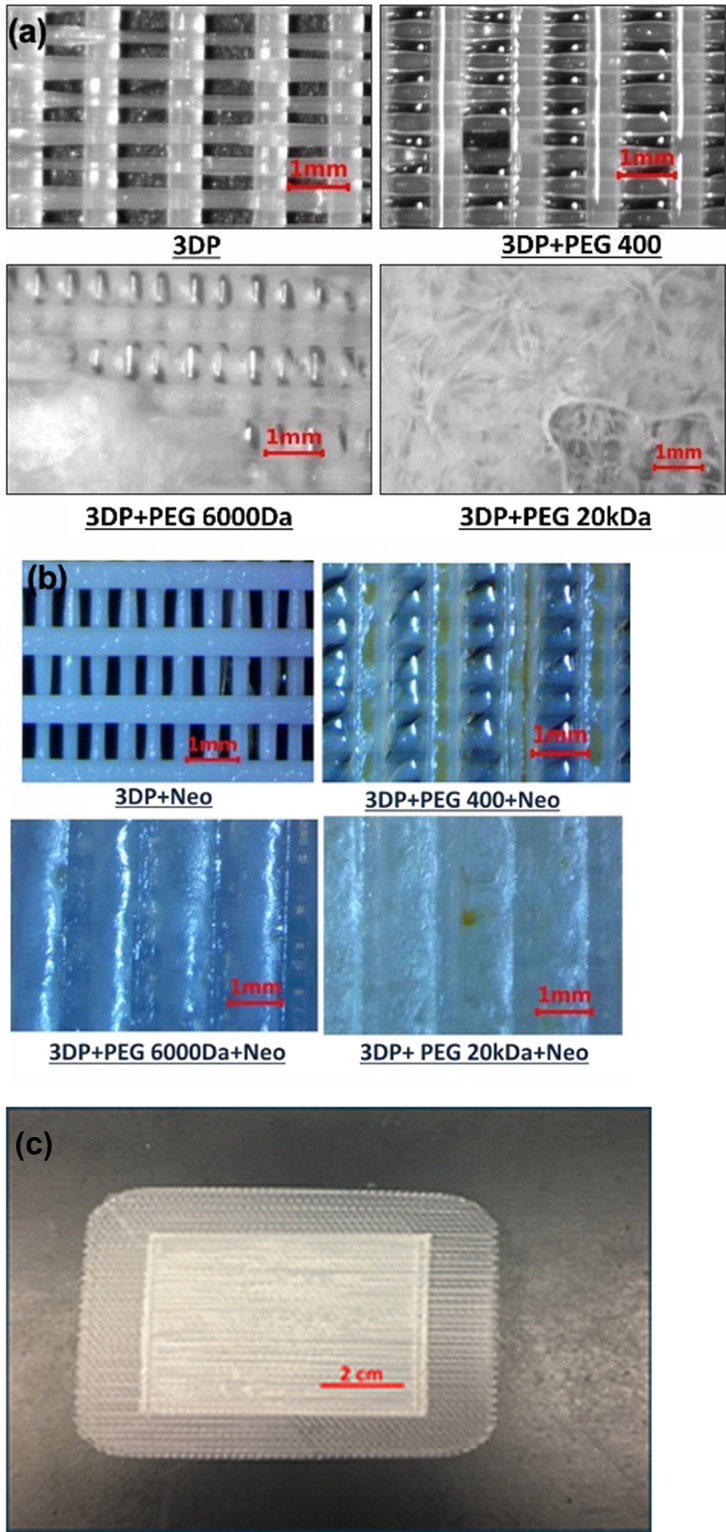
Polarized optical microscopic images of 3D-Printed (3DP) mats after coating with (**a**) PEGs, (**b**) PEGs + neomycin trisulfate, (**c**) Macroscopic view of the 3D printed mat

Figure 2a shows visual evidence of phase separation between PLLA and PEGs 6 and 20 kDa, but not for PLLA:PEG 400 Da. It should be noted that the visual evidence of macro-phase separation does not preclude mixing of PLLA and PEG at the molecular level. Previous studies have demonstrated that the solid-state miscibility of PEG in PLLA is about 12–20% w/w [60]. The presence of a yellow stint in Fig. 2b shows visual evidence of neomycin in these mats. A macroscopic view of the mat is shown in Fig. 2c.

**Fig. 3:**
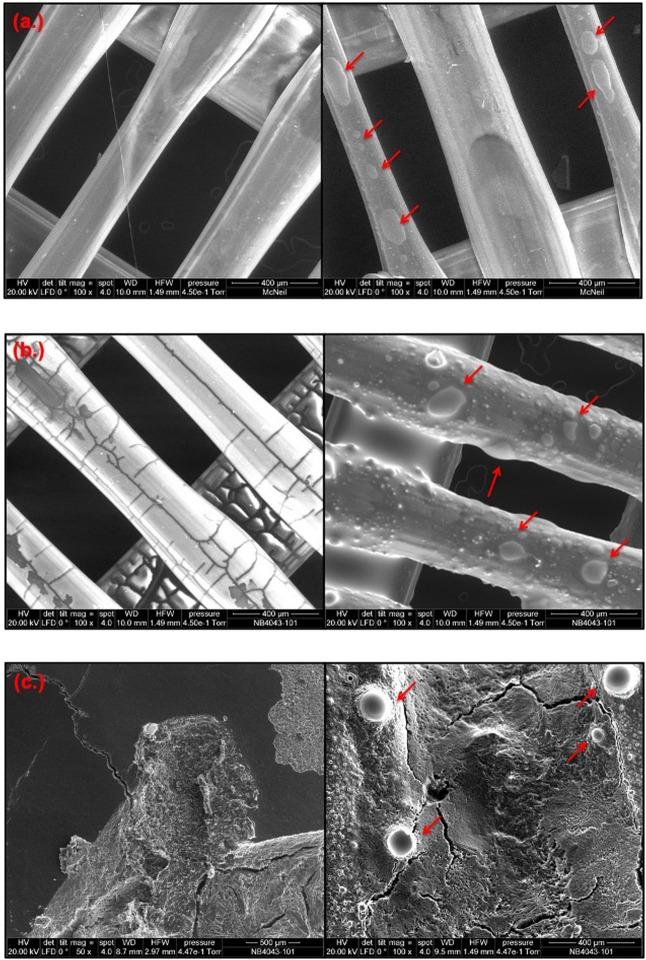
SEM images of surface of (**a**) 3DP mat, (**b**) 3DP + PEG 400 Da, and (**c**) 3DP + PEG 20 kDa (left side) along with neomycin-loaded samples (right side). Arrows indicate presence of neomycin. (PEG 6 kDa had appearance similar to 3DP + PEG 20 kDa and is not shown)

SEM images of the 3DP and 3DP + PEG 400 samples (Fig. 3a, b) appeared as perpendicularly aligned fibers, spaced to allow rectangular pores of dimensions 100 µm × 400 µm. Mats loaded with neomycin showed irregular patches (Fig. 3a). Coating with PEG 400 Da was uniform when compared to the flaky, nonhomogeneous appearance for PEG 6 and 20 kDa which showed several cracks.

### Mechanical strength

**Fig. 4:**
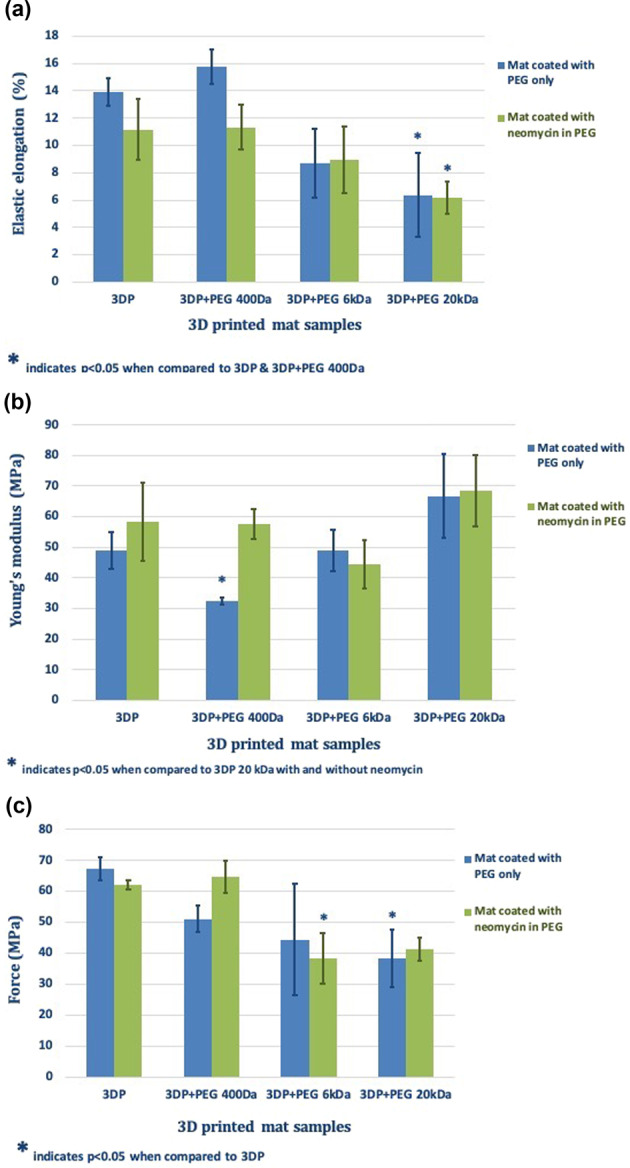
**a** Graph comparing the elastic elongation of all samples coated with three different molecular weights of PEG with or without neomycin loading. **b** Graph comparing the calculated Young’s modulus for all samples coated with three different molecular weights of PEG with or without neomycin loading. **c** Image showing the tensile strength comparison of all samples coated with three different molecular weights of PEG with or without neomycin loading

Figure 4a shows the percent EE of the fibers of all samples used to prepare coated and uncoated PLLA mats. The EE was relatively small and ranged from ~6 to 16%. Uncoated PLLA fibers, along with PEG 400-coated fibers showed the highest % EE. An inverse relationship between the MW of PEG used and % EE could also be observed from Fig. 4a. The inclusion of neomycin lowered % EE for uncoated and PEG 400 Da coated PLLA fibers, but not for those coated with PEG 6 and 20 kDa.

Figure 4b shows the Young’s modulus of the coated and uncoated PLLA fibers. The results appear to be opposite to the % EE observations. Uncoated and PEG 400 Da coated PLLA showed among the lowest Young’s modulus, while PLLA fibers coated with PEG 20 kDa had the highest Young’s modulus. The strength of the individual fibers ranged from 30 to 70 MPa (Fig. 4c). Both values were considered adequate for the intended application. The strength of the mat would be several orders of magnitude higher, because the overall strength would be a cumulative sum of all the fibers aligned in the direction of the applied stress.

### Differential scanning calorimetry

**Fig. 5:**
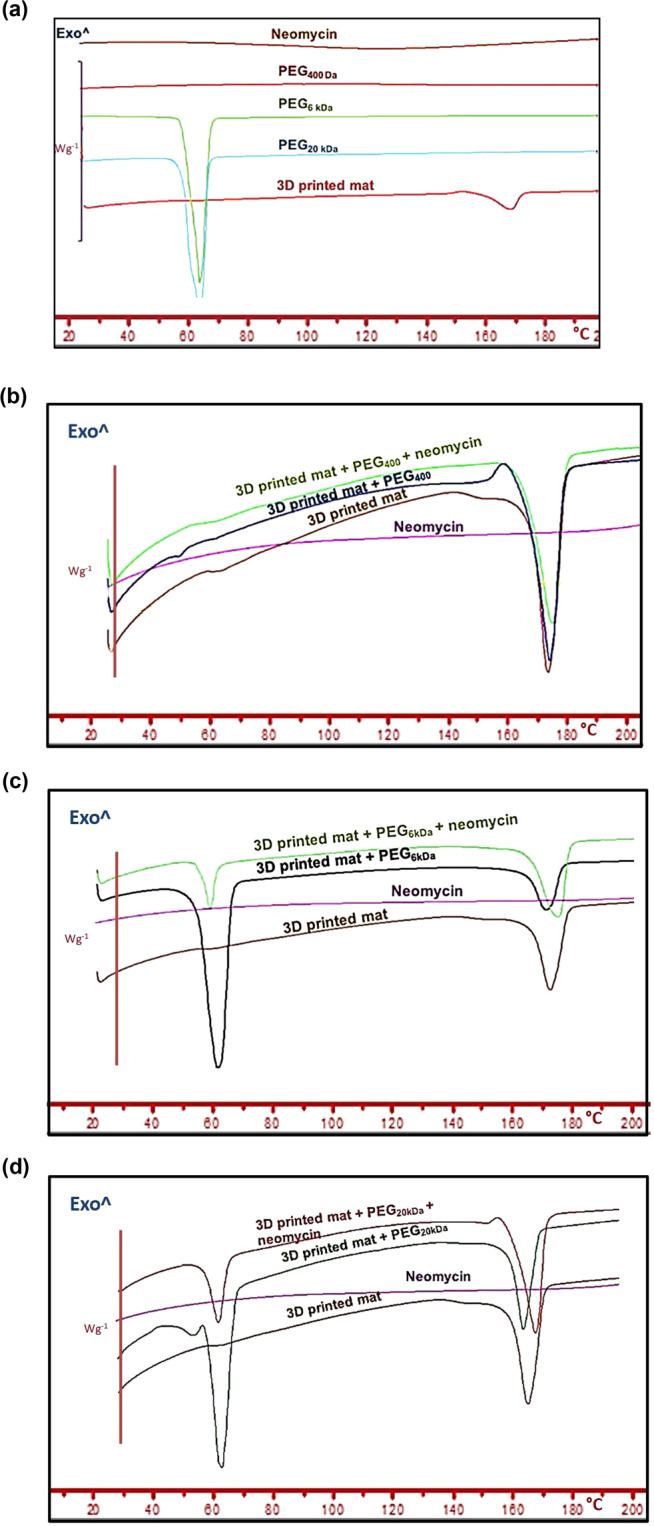
**a** Differential Scanning Calorimetry (DSC) thermograms of neomycin, PEG 400 Da, 6 kDa, 20 kDa, and 3DP PLLA mat. **b** Effect of PEG 400 Da on DSC thermograms of PLLA mats with and without neomycin trisulfate. **c** Effect of PEG 6 kDa on DSC thermograms of PLLA mats with and without neomycin trisulfate. **d** Effect of PEG 20 kDa on DSC thermograms of PLLA mats with and without neomycin trisulfate

Figure 5 shows DSC thermograms of 3DP mats prepared from PLLA, neomycin, and PEGs of three different MW. PLLA is a semicrystalline polymer, and is known to show both a glass transition as well as a melting temperature. Uncoated 3DP mats showed *T*_g_ at 62 °C and melting peak around 168–170 °C. PEGs are crystalline hydrophilic excipients, and solid PEGs are known to show a *T*_m_ between 60 and 65 °C [61]. PEG 400 Da is a liquid at room temperature, and therefore no transitions could be observed for this excipient in Fig. 5a. The absence of thermal transitions for neomycin suggests that this drug exists in its amorphous form. Figure 5b, c, d shows the effect of including PEGs of MW 400 Da, 6, and 20 kDa, respectively, on the solid-state phase transitions of PLLA mats with and without neomycin.

**Fig. 6:**
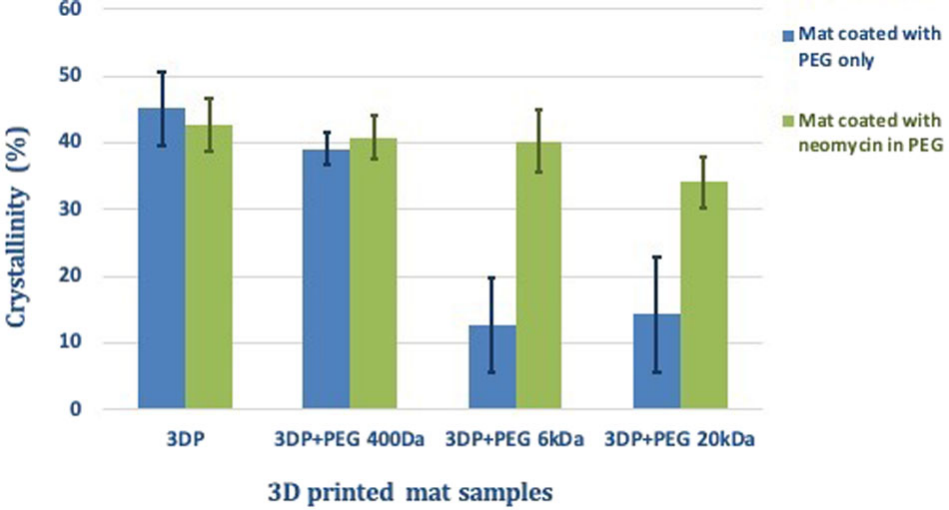
Image showing effects on crystallinity of 3DP PLLA mats when coated with PEG and loaded with neomycin

In the DSC thermograms shown in Fig. 5b–d, all phase transitions were observed in the individual thermograms of pure excipients. There were no significant shifts in PLLA or PEG melting peaks for mats were coated with PEG and loaded with neomycin. The *T*_g_ of 3DP mats was masked by melting enthalpies of PEG and could not be compared. To evaluate the effect of PEG coating and neomycin loading on the crystallinity of the 3D mats, the changes in the melting enthalpy values of the PLLA mat were evaluated as percent crystallinity. The Δ*H*_m_ was compared to a reference value of 93.7 J/g [55], which corresponds to literature value of 100% crystalline PLLA. The results are summarized in Fig. 6.

Figure 6 shows that the % crystallinity of PLLA in the mats ranges between 40 and 50%. No distinguishable differences could be observed between the % crystallinity of uncoated and 3DP + PEG 400 Da mats, with or without neomycin. However, 3DP mats coated with PEG 6 and 20 kDa showed a significant decline in crystallinity. This data correlated well with the tensile strength measurements (Fig. 4c), suggesting that crystallinity is important for the mechanical strength of these mats, and that the coating with PEGs of higher MW (6 or 20 kDa) lowers this strength.

### Powder X-ray diffractometry

**Fig. 7:**
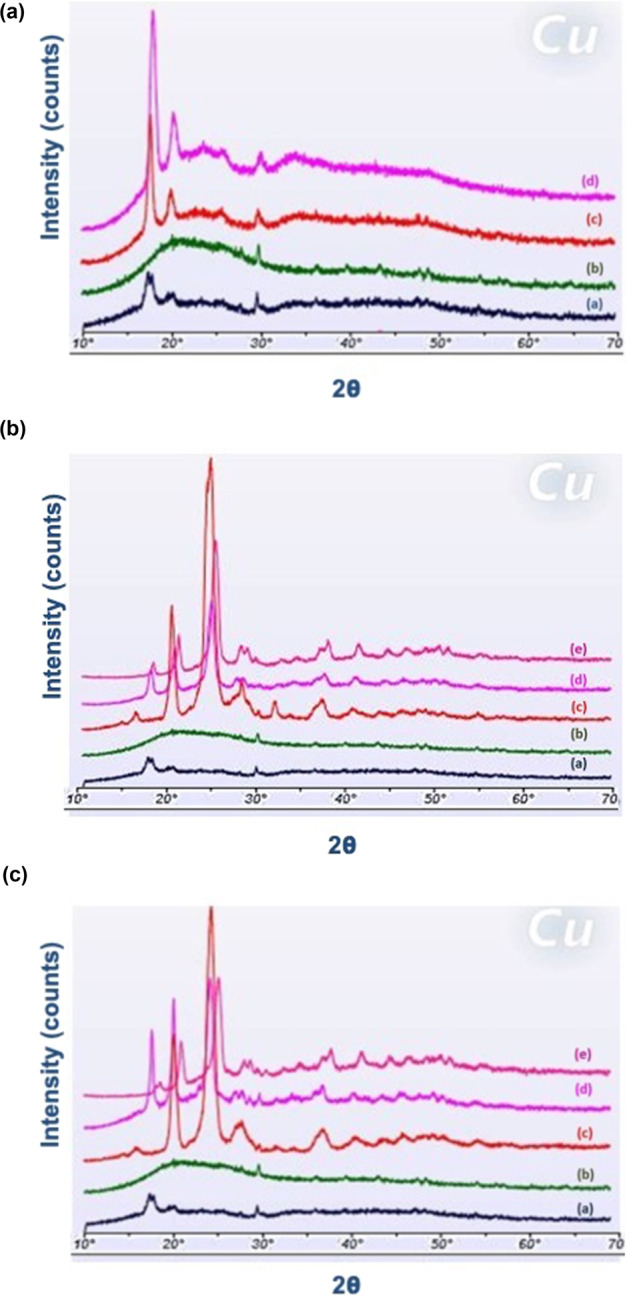
**a** X-ray diffractometer image showing (a) 3DP mat, (b) neomycin, (c) 3DP mat coated with PEG 400 Da, and (d) 3DP mat coated with PEG 400 Da and neomycin. **b** X-ray diffractometer image showing (a) 3DP mat, (b) neomycin, (c) PEG 6 kDa (d) 3DP mat coated with PEG 6 kDa, and (e) 3DP mat coated with PEG 6 kDa and loaded with neomycin. **c** X-ray diffractometer image showing (a) 3DP mat, (b) neomycin, (c) PEG 20 kDa (d) 3DP mat coated with PEG 20 kDa, and (e) 3DP mat coated with PEG 20 kDa and loaded with neomycin

Figure 7a, b, c show x-ray diffractograms of 3DP mats coated with PEG 400 Da, PEG 6 kDa, and PEG 20 kDa respectively. The diffractograms of pure PLLA mats showed crystalline peaks at 17.5° and 20.1°. The neomycin diffractogram was consistent with the DSC results, and it did not show crystalline peaks, but rather a broad amorphous halo between 10°and 30°. For the PEG 400-coated 3DP mats (Fig. 7a), no contribution was expected from PEG 400, which is known to be a liquid at room temperature. However, the crystalline peaks at 17.5° and 20.1° were much more prominent in PEG 400-coated 3DP, both with and without neomycin.

PEGs of MW 6 kDa and PEG 20 kDa are characterized by several large crystalline peaks, including one that overlaps with the 20.1° peak of PLLA. The coating process and inclusion of 3DP mats with PEG caused increased crystallization of PEG as evident in the larger crystalline PEG peaks between 23° and 28°. In Fig. 7b, c, only the first PLLA peak of 17.5° was considered for comparison to 3DP mats. An evaluation of these peaks showed a peak of intensity similar to 3DP mats, in the neomycin-loaded 3DP mats. 3DP mats coated with PEGs have a higher peak intensity at the 17.5° . These results are consistent with the mechanical testing data reported in Fig. 4a–c, which showed a significant decline in the %EE and tensile strength for corresponding 3DP mats.

Using Scherrer’s equation, crystal sizes *L*_hkl_ (nm) were compared with respect to the angular values [58]. A detailed evaluation of crystallite sizes is presented (Table 2). At 17.5° the crystal size of 3DP mat sample increases from 10.8 to 19.8 nm when coated with PEG 20 kDa. The crystalline size increases with increasing MW of PEG as well as with samples loaded with neomycin. The crystal size of 3DP sample remains the same when coated with PEG 400 Da and loaded with neomycin. However, for all other samples, it is increased in the presence of PEG with a direct proportionality to increase in MW of PEG, with and without neomycin. These results confirm the findings from crystallinity % as well as mechanical strength analysis where higher MW PEG change the crystalline behavior of 3DP mats whereas PEG 400 Da with and without neomycin does not affect the overall tensile strength of 3DP mats.

**Table 2:** A list of crystal sizes with respect to the angular values calculated using Scherrer’s equation

Sample	2θ	*d* (Å)	*β* (2θ)	*L*_hkl_ (nm)
3DP	17.5	5.06	0.73	10.8
	20.40	4.42	0.43	18.4
PEG_6 kDa_	20.14	4.41	0.58	13.8
	24.60	3.62	1.05	7.7
	28.18	3.16	1.29	6.3
PEG_20 kDa_	20.22	4.39	0.63	12.7
	24.62	3.61	0.93	8.7
	27.98	3.19	1.13	7.2
3DP + PEG_400_	17.5	4.99	0.51	15.5
	20.40	4.43	0.53	15.1
3DP + PEG_6 kDa_	17.5	4.98	0.51	15.5
	20.46	4.34	0.51	15.6
	25.74	3.46	0.26	30.1
	28.00	3.15	1.03	7.9
3DP + PEG_20 kDa_	17.5	5.00	0.40	19.8
	20.00	4.38	0.39	20.4
	24.62	3.65	0.71	11.4
	28.10	3.17	0.34	23.9
3DP + Neo	17.5	5.02	0.45	17.7
	20.00	4.42	0.82	9.7
3DP + PEG_400 Da_ + Neo	17.5	4.92	0.73	11.0
	20.46	4.35	0.68	11.7
	24.62	3.75	1.32	6.1
3DP + PEG_6 kDa_ + Neo	17.5	4.90	0.39	20.6
	20.94	4.24	0.43	18.4
	25.18	3.53	0.76	10.7
	28.10	3.17	0.34	23.7
3DP + PEG_20 kDa_ + Neo	17.5	4.75	0.41	19.2
	21.02	4.22	0.62	12.9
	25.38	3.51	0.80	10.1
	28.98	3.08	0.18	44.1

### In vitro neomycin dissolution

**Fig. 8:**
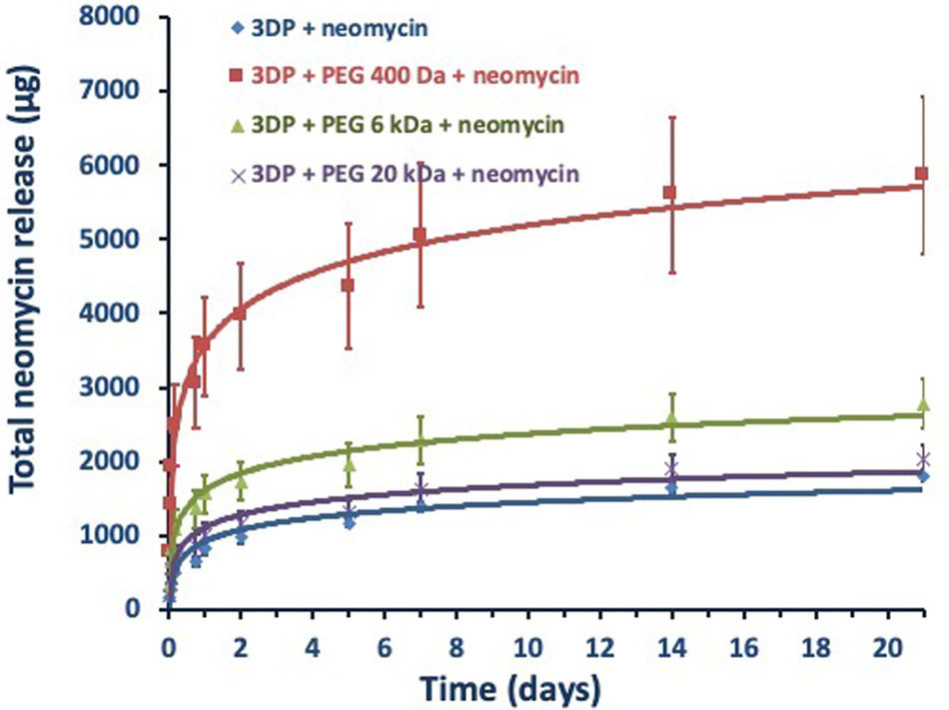
Total in vitro neomycin dissolution from 3DP mats coated with different molecular weights PEG coatings

Figure 8 shows the cumulative release of neomycin from the coated and uncoated 3DP mats, measured over 3 weeks. The results show that the neomycin release mechanism and duration was identical for all 3DP mats, and was unaffected by PEG coating or PEG MW. However, the total cumulative amount of neomycin released into the dissolution varied significantly, following an inverse relationship with the PEG MW. These results suggest that the use of higher MW PEGs lowers the drug loading potential for 3DP mats.

In summary, the results show that all 3DP mats coated with PEG 400 Da and neomycin had the highest % EE and a low young’s modulus, while showing the highest cumulative release of neomycin, distributed over a 3-week duration. The ability to maintain a high level of flexibility and drug loading make these mats the optimal choice for further devlopment.

## Discussion

This research used a synthetic, biodegradable semicrystalline polymer to prepare mats that could be potentially used as bandages. Our research established that a 3D printer equipped with a hot melt extruder may be used to prepare porous PLLA mats that can be subsequently coated with excipients, as well as loaded with a drug such as neomycin which can be released from the mat over a period of 3 weeks.

PLLA shows high tensile strength but tends to be stiff and brittle [62]. A higher % EE and lower young’s modulus of the samples, relative to 3D PLLA fibers is desired, as they afford flexibility to these fibers, without causing rupture. In previous studies, the inclusion of PEG has been shown to cause relevant surface, geometrical and structural changes causing modulation of chain mobility in PLA-based 3DP scaffolds [63, 64]. The use of lower molecular weight PEGs have been reported to show higher miscibility, and therefore cause favorable changes in the mechanical properties of PLA [65]. The results from our mechanical testing is consistent with these findings, whereby 3DP mats coated with PEG 400 Da showed greater % EE, lower Young’s modulus, and greater tensile strength compared to those with PEG 6 or 20 kDa. Similar effects of plasticity by PEG 400 Da and lowered Young’s modulus have been reported previously [66, 67]. The miscibility and low MW enables the molecules of PEG 400 Da to diffuse into the spaces between amorphous PLLA matrix, increasing free volume and enhance molecular mobility via greater rotation [68]. In the glassy solid state of the polymer, the molecular motion is restricted to vibrational and short-range rotational movement. The instability of higher MW PEGS has also been reported in PLA:PEG devices, and attributed to partial crystallization of PEG on cooling [69, 70]. It is possible that the large molecular size of PEG 6 and 20 kDa does not allow easy incorporation into the PLLA matrix.

The 3DP PLLA mat is a polydisperse system with crystallites of various sizes and shapes. Variations in the size of the crystallites typically results from the preparation and treatment-history of the sample [71]. Smaller crystalline grains can have simple geometric shapes such as spherical, cuboidal, tetrahedron, and octahedron while others can have more complex structures as cylinders, prisms or ellipsoids [72–75]. The diffractometer applies Bragg’s law (*nλ* = 2*d*sinθ) to measure diffraction pattern of constructive interference of diffracted light by the crystalline structures. The diffractogram provides information about crystallite-shape and sizes. The variations in the physical properties can be explained using the Scherrer’s equation. Using Scherrer’s equation (*L*_hkl_ = 0.89λ/*β*cosθ) [76], the different sizes of the crystallites in a polydisperse system can be analyzed [58]. We examined XRD patterns to determine the effect of PEG and neomycin coating onto PLLA mats. Our results indicate that measurements at 17.5° do not interfere with crystalline PEG peaks, and could be used to evaluate the effect of PEG and neomycin coating on the PLLA crystallites. The results showed that the d-spacing between the PLLA crystallites was uniform and varied between 4.75 and 5.06 Å. The crystallite size (*L*_HKL_) was also relatively uniform, and varied from 10.8 to 20.6 nm.

Neomycin is a commonly used topical antibiotic, with a reported aqueous solubility of 50 mg/ml. The permeation of this relatively water-soluble antibiotic could be facilitated by hydrophilic PEGs, which also have a high degree of miscibility with the PLLA matrix. Cumulative drug-release studies show that the coated 3DP matrices follow a proportionality with PEG MW, with PEG 400 Da showing the maximum release within a 3-week period. Mechanistic considerations in drug-release can vary depending on numerous factors such as dissolution, diffusion, partitionaning, osmosis, polymer swelling, and polymer surface or bulk erosion [77]. One of the most commonly used approach for release rate modeling is the higuchi model, which describes drug release from solid polymer matrix systems [78–80]. Higuchi model is valid over the time of duration of the dissolution given that the sink conditions are maintained, and is described by the equation:

$$f1 \,=\, Q \,=\, \sqrt {\frac{{D\varepsilon }}{\tau }\left( {2C \,-\, \varepsilon Cs} \right)Cst}$$

**Fig. 9:**
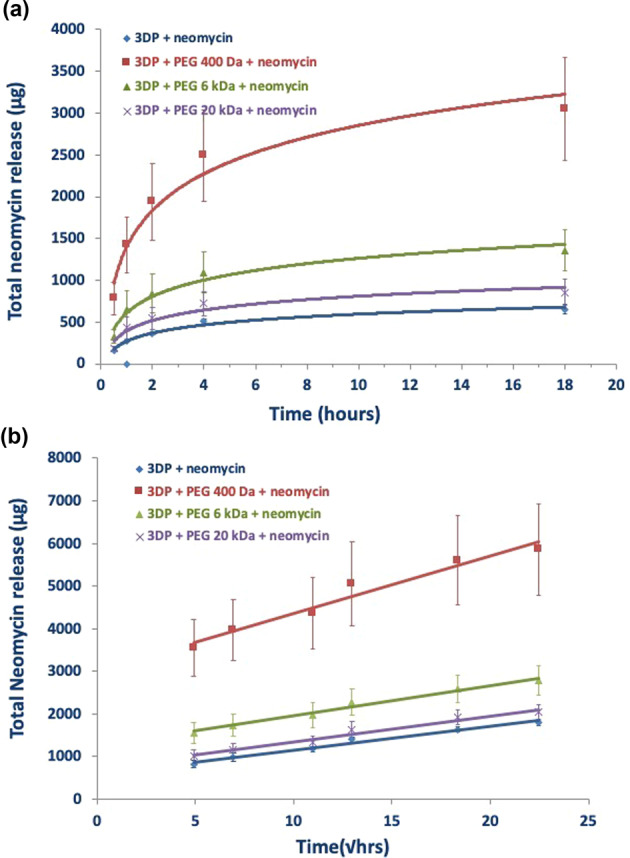
**a** Graphical representation of first-order release of neomycin over the first 20 h. **b** Graphical represention of cumulative neomycin release as a functin of square root of time showing linear release

Where *Q* = amount of drug released at time *t*, *ε* is the matrix porosity, *τ* is the capillary tortuosity factor (polymer chain relaxation time), *C* initial drug amount, *C*_s_ drug solubility, and *D* is the diffusion coefficient in the matrix medium. A further evaluation of the drug-release data showed a logarithmic fit with *r*^2^ > 0.93 indicating first-order release of neomycin from zero to 20 h (Fig. 9a), followed by diffusion-controlled (Fickian) release for the remaining duration of the study. A linear relationship with *r*^2^ > 0.96 between the cumulative amount of drug released versus the square root of time signifies a diffusion-controlled drug release as seen in Fig. 9b.

## Conclusions

Our study confirmed that a combined 3D printing, coating, and soaking method of mat preparation is a feasible option to prepare dermal mats. The impact of processing related stresses and change in partial crystallinity of a widely used polymer poly-l-lactic acid (PLLA) and its physical blend with polyethylene-glycol (PEG) was explored. Degradable polymer scaffolds were extruded and printed using a hot melt-extrusion technique in 3D printing. Results of thermal, mechanical, drug release, and imaging analysis assisted in characterizing scaffold properties for potential applications as a drug releasing mat

Morphological analysis showed porous structure suitable for cellular attachment. Mechanical testing showed formation of a flexible mat with PLLA + PEG 400 Da while higher MW PEGs made the PLLA mat “stiffer”. Addition of PEG, incorporated itself into the polymer chains of PLLA and spaced them apart increasing free volume. The presence of neomycin increased the miscibility of PEG into 3DP mats which means more of the PEG may be getting incorporated with the PLLA. The drug release from PEG 400 Da showed controlled release via passive diffusion. Overall, this study established that a 3DP, neomycin-loaded PLLA mat can be prepared with sufficient porosity and mechanical strength for applications as a dermal mat.
